# Does “crisis-induced intermittency” explain bipolar disorder dynamics?

**DOI:** 10.3389/fncom.2013.00116

**Published:** 2013-08-23

**Authors:** Fatemeh Hadaeghi, Mohammad R. Hashemi Golpayegani, Keivan Moradi

**Affiliations:** Biomedical Engineering Faculty, Amirkabir University of TechnologyTehran, Iran

**Keywords:** bipolar disorder, chaos theory, crisis, intermittency, mathematical modeling, multisatability, strange attractor

The brain presents a large number of spatially connected and interacting neurons and synapses that form many positive and negative feedback circuits. These complex networks in interaction with the environment have been experimentally demonstrated to produce temporally chaotic behavior which may be detected in recordings from individual nerve cells or neural ensembles (Korn and Faure, 2003). According to such paradigm, the brain could be considered as a complex system with chaos as its predominant dynamics. As a result, concepts of complex system and chaos theory could be applied to the studies of normal and abnormal brain functions.

One of the fundamental features of some complex systems is “multistability,” which can be understood as the coexistence of several interacting attractors (Chian et al., 2006). These interactions results in various complex behaviors in the long term dynamics of the system. Previous studies in several research areas, including neuroscience, have already reported the existence of multistability in natural systems (Chian et al., 2006; Goldbeter, 2011; Rabinovich et al., 2012).

From the perspective of chaos theory, irregular alternation between episodes of various forms of chaotic or periodic behaviors is known as “intermittency” (Tanaka et al., 2005; Chian et al., 2006). In a “global bifurcation,” an “attractor-merging crisis” could yield to intermittent behavior. This crisis occurs through the collision of two or more attractors with the boundaries of the basin of the attraction of other attractors (Tanaka et al., 2005; Chian et al., 2006). In this case, by crossing the boundary, the trajectory of the system would be attracted by the other attractor. Such trajectory would then, remain there until another crossing which may lead to a returning to the first attractor. Chaotic intermittency has been reported in circuit oscillators, economic variables, non-periodic associative dynamics in chaotic neural networks as well as in psychiatric disorders like obsessive–compulsive disorder (Tanaka et al., 2005; Chian et al., 2006; Rabinovich and Varona, 2011). However, we believe that such concept also could be applied to mood variation pattern in bipolar disorder.

According to physiological studies, neuroplastic variations may be the underlying mechanism which explain the misregulation of the main circuits involved in the emotional processing (Kandel et al., 2000; Berns and Nemeroff, 2003). This emotional dysregulation is somatically represented as irregular mood swings. Therefore, we believe that the clinical course of bipolar disorder, which is characterized by repeated erratic cycles of mania, depression and episodes of randomly appeared chaotic transitional states (Gottschalk et al., 1995; Berns and Nemeroff, 2003; Rabinovich et al., 2012), may also be understood based on the concept of chaotic intermittency. Manic, depressive and transitional states could be considered as stable or unstable attractors of a dynamical system through which the mood trajectory moves. Therefore, such accidental and abrupt changes of the mood state in bipolar disorder can result from the collision of the initial mood trajectory with the boundary of the basin of the attraction of the another mood attractors. According to chaos theory, this intermittent behavioral pattern could be considered as “crisis-induced intermittency.” Following such viewpoint, in healthy subjects, there would be only one “strange attractor” related to the mood states. Time series of such strange attractor represents both positive and negative emotions, unpredictably and in response to internal (for example thought, attention and memory) or external (environment) stimulus. In a bipolar person, however, initial emotional trigger of disease results in a type of “exterior crisis” in the system, in which the destruction of strange attractor is accompanied with formation of two abnormal attractors (mania and depression) and chaotic transients between them.

**Figure 1:**
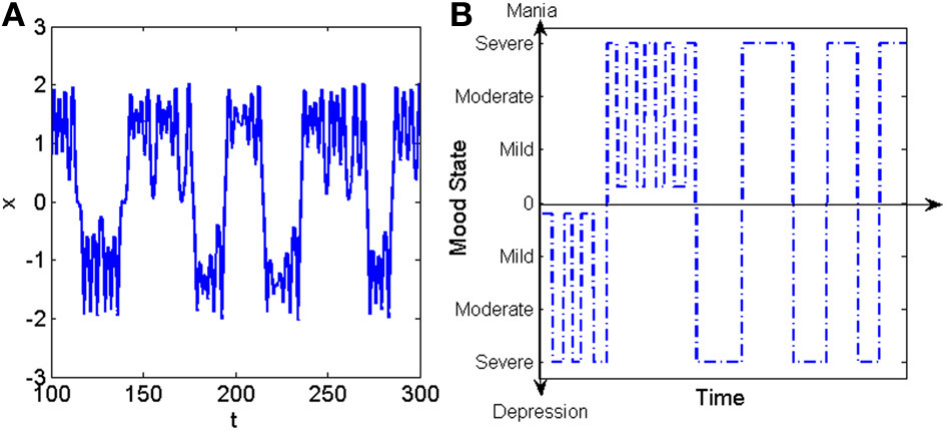
**(A)** Example of crisis induced intermittency in the forced Duffing oscillator. **(B)** Example of temporal pattern of mood variation in a patient with bipolar disorder (Tretter et al., 2011).

In order to model such scenario, models of chaotic systems which demonstrate various kind of crisis by changing their parameters (such as “forced Duffing” oscillator and “Ikeda” iterated map), could be utilized to characterize the basic features of human emotional states, when they are presenting multistable and intermittent behaviors, as in the case of bipolar disorder. In order to provide a deeper insight in to such dynamics, we represent the time series of forced Duffing oscillator in its crisis-induced intermittent mode in Figure 1A and an example of temporal pattern of self-rated mood records (life charts) in a person with bipolar disorder in Figure 1B. The proposed theoretical model would be useful in order to predict the evolution of such emotional states in bipolar disorder and to investigate the effects of psychopharmacological therapies. The experimental data for such investigations would most likely come from psychological tests, life chart recordings, or functional studies, such as EEG, fMRI, or PET-scan.
